# Correction to: FlowerNet: A Gene Expression Correlation Network for Anther and Pollen Development

**DOI:** 10.1093/plphys/kiad602

**Published:** 2023-11-14

This is a correction to: Simon Pearce, Alison Ferguson, John King, Zoe A. Wilson, FlowerNet: A Gene Expression Correlation Network for Anther and Pollen Development , Plant Physiology, Volume 167, Issue 4, April 2015, Pages 1717–1730, https://doi.org/10.1104/pp.114.253807

The FlowerNet community resource referenced in the originally published version of this manuscript is now available at http://www.cpib.ac.uk.

These details have been given in this correction notice to preserve the published version of record.

